# Supplementary material for: Modification of Natural and Synthetic Zeolites for CO2 Capture: Unrevealing the Role of the Compensation Cations
Source: Materials (Basel). 2025 May 21;18(10):2403. doi: 10.3390/ma18102403 (PMC12113214; doi:10.3390/ma18102403)
Supplement: Supplementary file 1 [file materials-18-02403-s001.zip › materials-3558150-supplementary.pdf]

Table S1. Langmuir isotherm parameters for raw and modified natural zeolites.

Zeolite Sample	Theoretical copper load (%)	Ion Exchange time (h)	Langmuir Model	
			kl (dm^3/mg)	Q_{max} (mg/g)
NZ	0	0	0.232	61.73
NZ_1	2	4	0.168	70.3
NZ_2	10	4	0.161	56.3
NZ_3	2	12	0.082	64.9
NZ_4	10	12	0.320	73.5
NZ_5	0.34	8	0.277	60.2
NZ_6	11.7	8	0.214	69.1
NZ_7	6	2.34	0.325	62.3
NZ_8	6	13.7	0.164	68.0
NZ_9	6	8	0.245	72.1
NZ_10	6	8	0.263	72.6
NZ_11	6	8	0.288	72.3
NZ_OPT	9.1	10.9	0.321	75.8

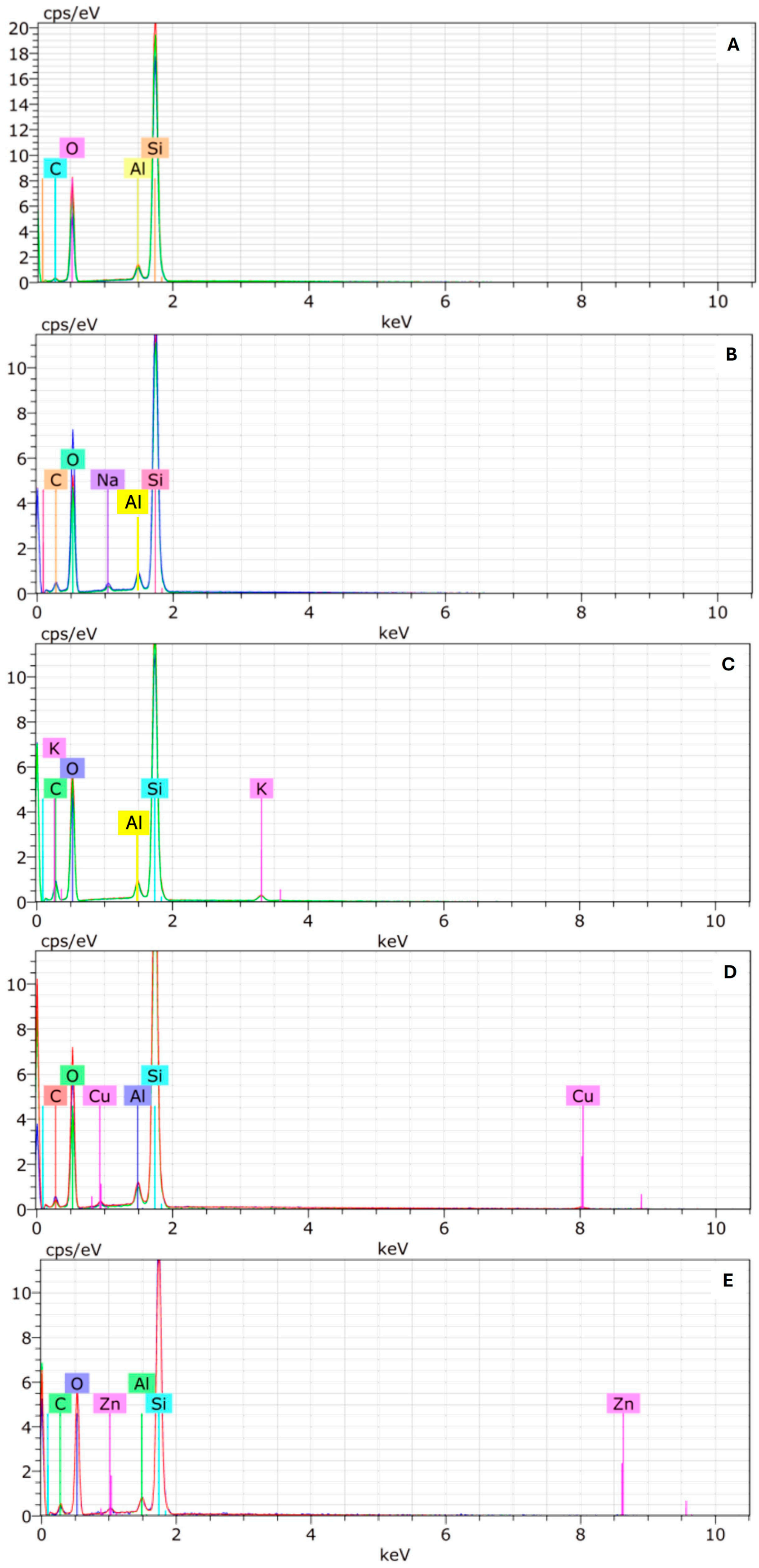

Figure S1: EDX Spectra of parent Synthetic Zeolite (**A**) and modified samples: SZ_Na (**B**), SZ_K (**C**), SZ_Cu (**D**) and SZ_Zn (**E**).

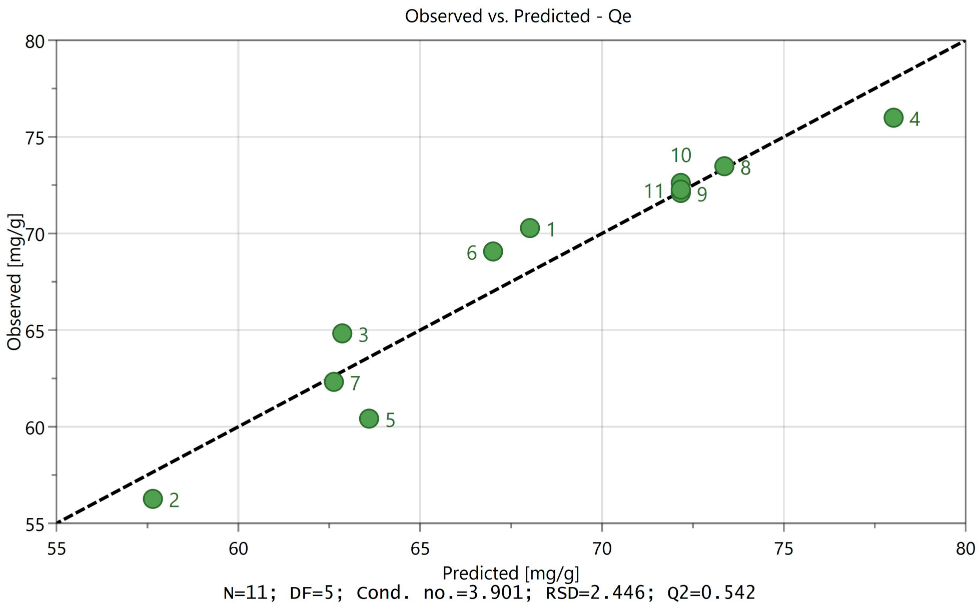

Figure S2. Values predicted from the quadratic model versus observed data.

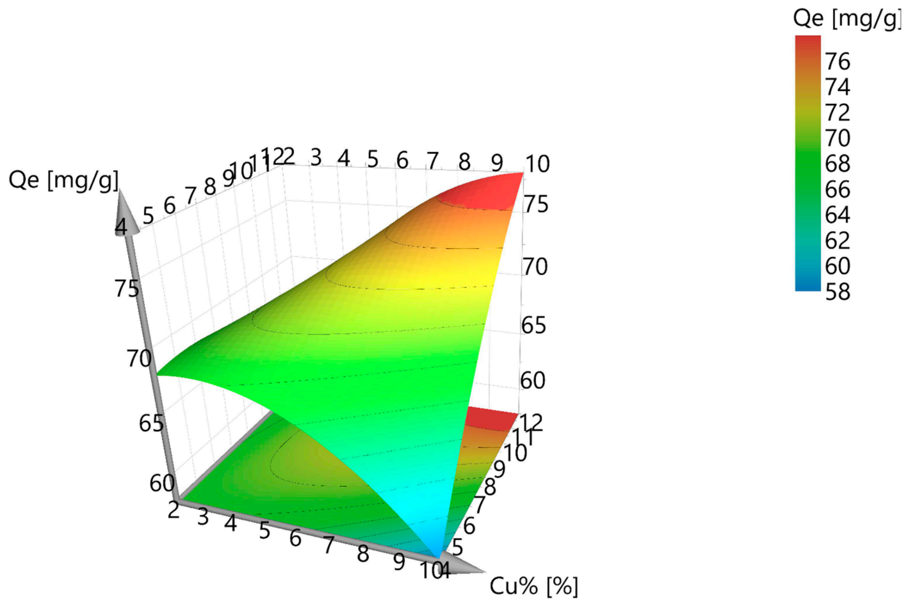

Figure S3. Response Surface Plot for CO_2 adsorption uptake onto modified natural zeolites.
